# Whole genome analyses of CMY-2-producing *Escherichia coli* isolates from humans, animals and food in Germany

**DOI:** 10.1186/s12864-018-4976-3

**Published:** 2018-08-09

**Authors:** Michael Pietsch, Alexandra Irrgang, Nicole Roschanski, Geovana Brenner Michael, Axel Hamprecht, Heime Rieber, Annemarie Käsbohrer, Stefan Schwarz, Uwe Rösler, Lothar Kreienbrock, Yvonne Pfeifer, Stephan Fuchs, Guido Werner, RESET Study Group, Anke Bühling, Burkhard Domurath, Christina von Salviati-Claudius, Constanze Wendt, Giuseppe Valenza, Hans Günther Wahl, Helene M. Lu, Henriette Laube, Johanna Hering, Josef Hellkamp, Mardjan Arvand, Michael Kresken, Rainer Podschun, Sandra Schneider, Steffen Tobisch, Marc Witt, Tim Eckmanns, Ulrike Hachmann, Uwe Bührlen

**Affiliations:** 1 Robert Koch-Institute, FG 13 Nosocomial Pathogens and Antibiotic Resistance, Burgstr, 37 38855 Wernigerode, Germany; 2 Department of Biological Safety, German Federal Institute for Risk Assessment (BfR), Berlin, Germany; 3 Freie Universität Berlin, Institute for Animal Hygiene and Environmental Health, Berlin, Germany; 4 Institute of Microbiology and Epizootics, Freie Universität Berlin, Berlin, Germany; 5 Institute of Farm Animal Genetics, Friedrich-Loeffler-Institut (FLI), Neustadt-Mariensee, Germany; 6 Institute for Medical Microbiology, Immunology and Hygiene, University of Cologne, University Hospital Cologne, Cologne, Germany; 7 Medizinisches Versorgungszentrum Dr. Stein, Division of Microbiology, Mönchengladbach, Germany; 8 Veterinary University Vienna, Vienna, Austria; 9 Institute for Biometrics, Epidemiology and Data Processing, University of Veterinary Medicine, Hanover, Germany

**Keywords:** Multidrug-resistant, Food chain, Plasmid, AmpC beta-lactamase

## Abstract

**Background:**

Resistance to 3rd-generation cephalosporins in *Escherichia coli* is mostly mediated by extended-spectrum beta-lactamases (ESBLs) or AmpC beta-lactamases. Besides overexpression of the species-specific chromosomal *ampC* gene, acquisition of plasmid-encoded *ampC* genes, e.g. *bla*_CMY-2_, has been described worldwide in *E. coli* from humans and animals. To investigate a possible transmission of *bla*_CMY-2_ along the food production chain, we conducted a next-generation sequencing (NGS)-based analysis of 164 CMY-2-producing *E. coli *isolates from humans, livestock animals and foodstuff from Germany.

**Results:**

The data of the 164 sequenced isolates revealed 59 different sequence types (STs); the most prevalent ones were ST38 (*n* = 19), ST131 (*n* = 16) and ST117 (*n* = 13). Two STs were present in all reservoirs: ST131 (human *n* = 8; food *n* = 2; animal *n* = 6) and ST38 (human *n* = 3; animal *n* = 9; food *n* = 7). All but one CMY-2-producing ST131 isolates belonged to the clade B (*fimH*22) that differed substantially from the worldwide dominant CTX-M-15-producing clonal lineage ST131-O25b clade C (*fimH*30). Plasmid replicon types IncI1 (*n* = 61) and IncK (*n* = 72) were identified for the majority of *bla*_CMY-2_-carrying plasmids. Plasmid sequence comparisons showed a remarkable sequence identity, especially for IncK plasmids. Associations of replicon types and distinct STs were shown for IncK and ST57, ST429 and ST38 as well as for IncI1 and ST58. Additional β-lactamase genes (*bla*_TEM_, *bla*_CTX-M_, *bla*_OXA_, *bla*_SHV_) were detected in 50% of the isolates, and twelve *E. coli* from chicken and retail chicken meat carried the colistin resistance gene *mcr-1.*

**Conclusion:**

We found isolates of distinct *E. coli* clonal lineages (ST131 and ST38) in all three reservoirs. However, a direct clonal relationship of isolates from food animals and humans was only noticeable for a few cases. The CMY-2-producing *E. coli*-ST131 represents a clonal lineage different from the CTX-M-15-producing ST131-O25b cluster. Apart from the ST-driven spread, plasmid-mediated spread, especially via IncI1 and IncK plasmids, likely plays an important role for emergence and transmission of *bla*_CMY-2_ between animals and humans*.*

**Electronic supplementary material:**

The online version of this article (10.1186/s12864-018-4976-3) contains supplementary material, which is available to authorized users.

## Background

The production of extended-spectrum β-lactamases (ESBLs) is the worldwide most important mechanism of resistance to 3rd-generation cephalosporins in *Escherichia coli.* [1]. AmpC beta-lactamases are also able to hydrolyse 3rd-generation cephalosporins. Induction or overexpression of chromosomally-located, species-specific *ampC* genes and the acquisition of plasmid-encoded *ampC* genes (e.g. *bla*_CMY-like_, *bla*_ACC-like_, *bla*_DHA-like_) have been described in *E. coli* [2]. The most common plasmidic *ampC* gene reported in *Enterobacteriaceae* including *E. coli* is *bla*_CMY-2_ [3, 4]. It originates from the chromosomal *ampC* gene of *Citrobacter freundii,* and had been mobilized onto plasmids of different replicon types (IncK, IncI1, IncA/C and IncFIA-FIB) by the insertion sequence (IS) IS*Ecp1* that also provides the promotor for high-level expression of *bla*_CMY-2_ [2].

In recent years an increasing number of CMY-2-producing *E. coli* was noticed in the European livestock production. Especially in poultry, there is a prevalence of more than 30% among 3rd-generation cephalosporin-resistant *E. coli*, whereas only ~ 1% of the 3rd-generation cephalosporin-resistant *E. coli* from humans harboured the *bla*_CMY-2_ gene [3, 5, 6]. Recent studies reported the finding of *bla*_CMY-2_ on similar IncK or IncI1 plasmids in unrelated *E. coli* isolates from poultry and other livestock animals, meat products, humans and companion animals [7–10]. This finding points towards a zoonotic potential for the dissemination of this resistance determinant via the food production chain. In recent years, a big variety of clonal lineages of *E. coli* has been reported worldwide, some of them have proved to be dominant. Of interest are ESBL-producing *E. coli* of sequence type (ST)131 that are prevalent in humans but not in livestock animals and food [3, 11, 12]. Using sequence based analyses ST131 has been grouped into different clades, which are usually associated with specific *fimH* alleles: clade A (*fimH*41 ST131-O16), clade B (*fimH*22 ST131-O25b) and clade C (*fimH*30 including ST131-O25b *fimH*30-R/*fimH*30-Rx) [13, 14]. Especially strains of ST131-O25b with presence of ESBL-type CTX-M-15 and resistance to fluoroquinolones (*fimH*30-Rx) have been reported worldwide, and frequently represent a cause of infections, particularly urinary tract infections, in human patients [15, 16]. However, CMY-2 production has been hitherto rarely described for ST131 isolates [7, 17].

To investigate a possible transmission of *bla*_CMY-2_ along the food production chain, we conducted next-generation sequencing (NGS)-based analysis of CMY-2-producing *E. coli* isolates from humans, livestock animals and foodstuff from Germany.

## Results

### Antibiotic susceptibilities, resistance and virulence genes

All 164 CMY-2-producing *E. coli* isolates of our study were resistant to ampicillin, cefotaxime, ceftazidime and cefoxitin but remained susceptible to imipenem and meropenem with one exception (isolate no. 10–16 with non-susceptibility to imipenem and meropenem). Additional resistance to ciprofloxacin was detected in 25% (41/164) of the isolates. The proportion of resistance to ciprofloxacin in CMY-2-producing *E. coli* from humans was 43.5% (20/46) remarkably higher than with 17.8% (21/118) in isolates from livestock animals and meat products.

Presence of resistance genes in the whole genome sequences of the 164 *E. coli* isolates was investigated by ResFinder (Additional file 1: Table S1). All genomes contained *bla*_CMY-2_. Additional β-lacatamase genes were present quite frequently: *bla*_TEM-like_ (*n* = 72), *bla*_CTX-M-1/9-group_ (*n* = 8), *bla*_OXA-1-like_ (*n* = 5), *bla*_SHV-like_ (*n* = 5) and *bla*_OXA-10-like_ (*n* = 1). Several isolates carried plasmid-mediated quinolone resistance (PMQR) genes that contribute to ciprofloxacin-resistance: *aac(6′)-Ib-cr* (*n* = 7), *qnrS1* (*n* = 4), *qnrB1* (*n* = 1), and *qnrB19* (*n* = 1). Furthermore, the plasmid-mediated colistin resistance gene *mcr-1* was observed in twelve isolates from livestock animals (chickens *n* = 3; pigs *n* = 5; turkey *n* = 1) and food (n = 3). Subsequently performed susceptibility tests (broth microdilution test according to EUCAST criteria v. 7.1) confirmed resistance to colistin (MIC 4–8 mg/L) of these 12 isolates.

Additionally, the presence of shigatoxin gene *stx-2* was observed in one isolate (isolate no 6–16, serotype O141:H49).

### MLST analysis

Our collection of 164 *bla*_CMY-2_-positive *E. coli* isolates showed a high diversity of sequence types (ST). For isolates from human patients (*n* = 46), isolates from livestock animals (*n* = 63), and isolates from food (*n* = 55) we determined 31, 29, and 20 different STs, respectively (Table 1). The most prevalent STs were ST38 (*n* = 19), ST131 (*n* = 14) and ST117 (*n* = 14). In isolates from human patients the most prevalent ST was ST131 (*n* = 6; 13%), its proportion among isolates from livestock animals and food was 12.2 and 3.8%, respectively. However, in isolates from livestock animals and food products ST38 was most prevalent (chicken meat *n* = 9; chickens *n* = 6; cattle *n* = 1). Several STs were present in *E. coli* from all three sources: ST38 (humans *n* = 3; chicken meat n = 9; chickens *n* = 6; cattle *n* = 1), ST131 (humans *n* = 6; chicken meat *n* = 2; chickens *n* = 5; turkey *n* = 1), ST117 (human *n* = 1; chicken meat *n* = 5; turkey meat *n* = 1; chickens *n* = 7) and ST10 (humans *n* = 4; chicken meat n = 1; turkey meat *n* = 1; pig meat *n* = 2; chickens *n* = 1; pigs *n* = 1). In contrast, other STs, e.g. ST429, were only observed in chicken meat products and chicken isolates (*n* = 3/3) (Table 1).

**Table 1:** Multilocus sequence typing (MLST) of 164 CMY-2-producing *Escherichia coli* from different sources, 2008-2013, Germany

MLST *E. coli* human patients		MLST *E. coli* from food						MLST *E. coli* from livestock animals							
		chicken		turkey		pork		chicken		turkey		pig		cattle	
ST	*n*	ST	*n*	ST	*n*	ST	*n*	ST	*n*	ST	*n*	ST	*n*	ST	*n*
ST131	6 (13.0%)	ST38	9	ST10	1	ST10	2	ST117	6	ST1196	3	ST1196	3	ST88	1
ST10	4	ST58	5	ST5763	1	ST75	1	ST38	6			ST212	1	ST38	1
ST69	3	ST57	5	ST2040	1			ST131	5 (12.2%)			ST10	1		
ST38	3	ST117	5	ST117	1			ST3778	4			ST3778	1		
ST354	2	ST69	4	ST6008	1			ST429	3			ST57	1		
ST58	2	ST429	3	ST421	1			ST57	3			ST1594	1		
ST453	2	ST2040	3	ST1463	1			ST2040	3			ST2040	1		
ST1844	1	ST68	2					ST420	1						
ST59	1	ST131	2 (3.8%)					ST1158	1						
ST141	1	ST155	2					ST540	1						
ST744	1	ST569	1					ST1196	1						
ST224	1	ST1286	1					ST155	1						
ST4759	1	ST420	1					ST2309	1						
ST23	1	ST10	1					ST162	1						
ST117	1	ST4937	1					ST2458	1						
ST2450	1	ST1818	1					ST354	1						
ST448	1	ST115	1					ST10	1						
ST348	1	ST1594	1					ST212	1						
ST457	1	ST373	1												
ST86	1	ST108	1												
ST1463	1	ST752	1												
ST93	1	ST350	1												
ST648	1	ST354	1												
ST127	1														
ST694	1														
ST115	1														
ST2077	1														
ST393	1														
ST963	1														
ST355	1														
ST362	1														
Total	46		53		7		3		41		3		9		2

Proportion of the most frequent sequence type ST131 in the respective source is given in %

### cgMLST analysis

**Fig. 1:**
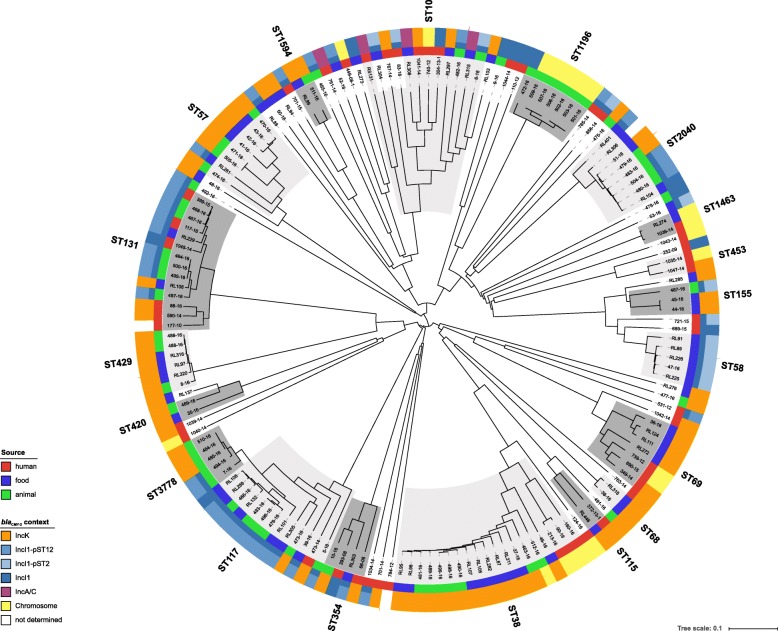
NGS-based neighbour-joining tree of 164 *E. coli* isolates based on an ad-hoc cgMLST including 2547 alleles. The tree was built with SeqSphere+ and visualized by iTOL v.3. Different MLST clusters are highlighted with dark/light grey shadows. The color-coded rings represent the origins of the samples (inner ring) and the corresponding replicon types to the present *bla*_CMY-2_ carrying plasmids (outer rings)

The application of a core genome multilocus sequence typing (cgMLST) scheme achieved a much higher resolution (comparison of 2547 alleles vs. seven MLST alleles) regarding the genetic relatedness of the CMY-2-producing *E. coli* isolates. As expected, isolates forming one ST by the classical multilocus sequence typing (MLST) scheme grouped together in the cgMLST scheme, but due to the higher discrimination capability of the cgMLST, subgroups within the respective cgMLST cluster were observed (Fig. 1). The highest diversity was observed for ST10 isolates; the allele differences between ST10s isolates varied between 326 and 982). In general, isolates from the same source (human, livestock animal or food) grouped more closely together, but also clusters of isolates from animals and meat products could be observed, e.g. ST117 and ST2040 (Fig. 1). Most isolates from human patients showed a noticeably higher allele distance to isolates from livestock animals and food. Exceptions were observed for ST131 and ST1463. ST131 isolates from the three sources grouped into mixed clades with an allele difference of 15–205. In contrast, ST38 isolates from human patients separated from the livestock/food isolates by a minimum of 513 alleles, whereas the difference between livestock and food isolates was 1–190 alleles. The most related isolates were identified in ST429 (livestock/food isolates, allele difference 0–46), ST117 and ST3778 (allele difference 0–79). ST117 and ST3778 differ in three nucleotide substitutions in the *gyr* allele (*gyr*4- > *gyr*14: 174C > T; 288C > T; 396 T > C), and therefore, form one group in the cgMLST scheme. All but one (isolate no. 473–14, human tracheal secretion isolate) isolate of these two STs were from livestock (chickens *n* = 10; pig *n* = 1) and food (chicken meat *n* = 5; turkey meat *n* = 1).

In an additional analysis we compared our ST429 isolates to all available ST429 *E. coli* isolates from EnteroBase (http://enterobase.warwick.ac.uk/). Our cgMLST analysis demonstrated that a cluster of *E. coli* isolates from chicken meat products and chicken from Denmark, Germany and France showed a high genetic relatedness (Additional file 2: Figure S1).

### SNP analysis

Isolates from selected cgMLST clusters (ST131, ST1196, ST429 and ST117/3778) were analysed by a single nucleotide polymorphisms (SNP)-based mapping approach. For each cgMLST cluster the best fitting reference for the mapping-based SNP analysis was identified using refRank. *E. coli* strain MDR_56 (GenBank accession no. NZ_CP019903.1) was determined as the best available reference for ST117/ST3778. The analysis of ST117/ST3778 isolates revealed three clusters with differences from 0 to 47 SNPs in each cluster (Additional file 3: Figure S2a**).** Notably, cluster A (ST3778) isolates no. 510–16 and 484–16 from different farms in the federal state of Brandenburg showed no SNPs (exclusion distance d = 0) and originated from chicken faeces (isolated in 2011) and an environmental sample from a pig farm (isolated 2011) within a distance of ca. 50 km. Another isolate (no. 485–16) from chicken faeces, isolated in 2011 in the federal state Saxony-Anhalt, represented a single allele difference and only four SNPs compared to isolates no. 510–16 and 484–16. Cluster B and C equally presented closely related ST117 isolates (3–20 SNPs in cluster B and 11–33 SNPs in cluster C observed) and originated from chicken (*n* = 4) and chicken meat (*n* = 1) and turkey meat (*n* = 1), isolated in 2012 in the federal states Lower Saxony and Hesse (Additional file 3: Figure S2a).

Within ST131, three closely related isolates from livestock in farms of different federal states (all isolated 2012) were observed: no. 464–16 (chicken, Lower-Saxony), no. 500–16 (turkey, Bavaria) and no. 495–16 (chicken, Bavaria) demonstrating 0 (no. 464–16 to no. 500–16) to 3 (no. 500–16 to no. 495–16) allele differences and 1 to 6 SNPs differences, respectively (Additional file 3: Figure S2b). Interestingly, two isolates from human patients (no. 399–15 and no. 1045–14; isolated in November 2013 and August 2014) differed by only 28 and 27 SNPs, respectively, from a chicken isolate (no. 468–16, isolated in August 2012). These three closely related isolates were collected in Western Germany (radius 200 km) within a period of three years indicating a trans-sectoral spread.

The seven identified isolates assigned to ST1196 represented closely related strains from livestock animals, varying from 0 to 125 allele differences in the cgMLST scheme and grouping into two clusters (Additional file 3: Figure S2c). Cluster A included three isolates from turkeys**.** These isolates were collected in three stables from two farms in the same federal state in 2014. Differences between the isolates were from 1 to 3 alleles and 0 to 3 SNPs (by mapping against for ST1196 isolates best determined reference NC_020518.1 *E. coli* str. K-12 substr. MDS42). Cluster B consisted of three isolates from pigs, isolated in the federal states North Rhine-Westphalia and Saxony-Anhalt in 2011, showing 0 allele and 0 to 2 SNPs differences. One additional isolate from chicken (no. 472–16) was sampled in 2011 in the same geographical region as the pig isolates (cluster B) but differed by 99 SNPs or by 88 alleles to cluster A isolates.

The two closely related ST1463 isolates from human and turkey meat, respectively, exhibited high similarity in the cgMLST approach (19 allele differences), and only 21 SNPs were detected after mapping (for ST1463 isolates best determined reference: GenBank accession no. NC_020518.1, data not shown). The isolates were identified in Bavaria and North Rhine-Westphalia in the years 2012 and 2014, respectively.

All *E. coli*-ST429 isolates were from livestock animals and food and showed only a small number of allele variants (Additional file 3: Figure S2d). Two isolates from chickens were collected in the same federal state within a 16 month period between 2011 and 2012 and showed 0 allele and 0 SNP differences. Isolates from chicken meat - which were recovered from meat products bought 2012 in Lower Saxony and Bavaria - presented only 6 and 8 alleles difference, respectively, to the other isolates of this ST from chicken. Furthermore, isolate no. 8–16 from a diseased chicken (isolated in 2012 in Bavaria) showed a slightly distant similarity (32 to 46 allele differences) to all other *E. coli*-ST429 isolates.

### *fimH* analysis of ST131-*E. coli* isolates

Typing of the *fimH* gene of *E. coli* can be used to elucidate the population structure within a ST and is used to differentiate the subclades of ST131 [12]. Our analysis showed that all but one CMY-2-producing ST131-*E. coli* (*n* = 14) belonged to the clade B, and were associated with *fimH*22. The exceptional isolate no. 177–10 (human origin) harboured *fimH*30 (clade C) instead and carried an additional ESBL gene *bla*_CTX-M-15_. Isolates belonging to clade A (*fimH*41) were not identified. Sequence data suggested a plasmid location of *bla*_CMY-2_ in isolate 177–10 but not on an IncI1 or IncK2 plasmid as found for most CMY-2 *E. coli* isolates in our study (see below). A screening of nearly 1000 available *E. coli-*ST131 sequences from the years 2008 to 2017 (sequences were obtained from the International Nucleotide Sequence Database Collaboration (INSDC) by the SS+ NCBI Bacteria Genome Browser and *E. coli* assemblies from EnteroBase (http://enterobase.warwick.ac.uk/species/index/ecoli)) revealed only 15 *bla*_CMY-2_-positive strains (Additional file 4: Figure S3). The majority (*n* = 9) of these 15 isolates clustered in the applied cgMLST scheme in clade B (*fimH*22), along with our study isolates; only one isolate harboured a *fimH*41 allele (clade A). The remaining five isolates carried *fimH*30 (clade C) but no additionally *bla*_CTX-M-15_ gene and IncK or IncI1 replicon sequences were present. In general, most of the compared ST131 isolates from EnteroBase contained *fimH*30 with *bla*_CTX-M-15_ (*n* = 377), *fimH*30 without *bla*_CTX-M-15_ (*n* = 287) or *fimH*30 with *bla*_CTX-M-27_ (*n* = 87). The *fimH* variants grouped exclusively according to their variants in the cgMLST schema. A total of 789 *fimH*30 isolates were identified, in addition to 66 *fimH*41, 16 *fimH*27 and 84 *fimH*22; most of the *bla*_CMY-2_-positive isolates cluster in the latter clade H22 (Additional file 4: Figure S3). Isolate no. 177–10 clustered together with *fimH*30 ESBL-positive strains in clade C.

### Plasmid analysis

In general, the most frequent replicon types identified by PlasmidFinder were FIB (*n* = 133), FII (*n* = 88), FIA (*n* = 33) and FIC (*n* = 55), I1 (*n* = 81) and B/O/K/Z (*n* = 78); further replicon types were A/C (*n* = 7), N (*n* = 10) and I2 (*n* = 12). Contig aligned to several completely assembled *bla*_CMY-2_-carrying plasmids from GenBank database enabled the identification of *bla*_CMY-2_ location.

#### IncK plasmids

In 72 *E. coli* isolates (human *n* = 14; livestock *n* = 25; food *n* = 37) *bla*_CMY-2_ has been linked to plasmids of the recently defined incompatibility group IncK2 [8] (Additional file 1: Table S1). In 26 isolates the link between *bla*_CMY-2_ and the IncK2 replicon sequence was confirmed on the same de novo assembled contig. In the remaining 46 isolates multiple occurrences of identical insertion sequences (IS) in the sequence data prevented a complete de novo assembly. In this case, a mapping of contigs to previously published *bla*_CMY-2_-carrying plasmid sequences (pTMSA1088 and pDV45) was conducted to deduce the respective plasmid contigs. Only one plasmid backbone with minor (SNPs) and major (mobile genetic element (MGE) insertions and variable shufflon region) alterations was identified among all IncK2 plasmid sequences. This backbone was identical to the sequence of the annotated and fully assembled *bla*_CMY-2_-carrying plasmids pDV45 (85.9 kb) and pTMSA1088 (79.3 kb). These two plasmids differed by a ca. 6.5 kb sized fragment inserted between the genes *yfbA* and *psiB* and presented a highly variable shufflon region [8]. We confirmed these plasmid sizes by S1 nuclease pulsed-field gel electrophoresis (PFGE) for selected isolates of this study. The Inc type was confirmed by PBRT [8].

**Fig. 2:**
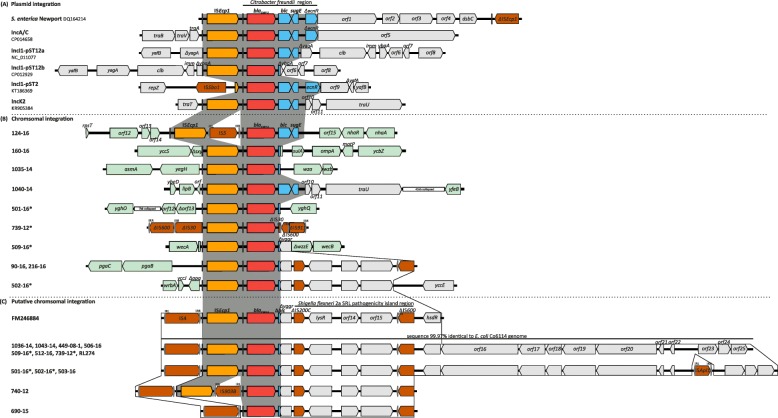
Surrounding genetic regions of *bla*_CMY-2_ in the 164 *E. coli* isolates from the different reservoirs. IS*Ecp1* and the adjacent from *Citrobacter freundii* mobilized conserved chromosomal region containing *bla*_CMY-2_, *blc*, *sugE* and *ecnR* is highlighted in dark grey across the different backgrounds. The color code is as follows: IS*Ecp1* is in orange, *bla*_CMY-2_ in red, *blc*, *sugE*, and *ecnR* are in blue, insertion sequence elements (IS) in brown, *E. coli* chromosomal genes adjacent the *bla*_CMY-2_ complex integration are in light green. The black rectangles represent the inverted repeats of IS*Ecp1*; further inverted repeats are highlighted by grey rectangles. **a** Genetic surrounding of *bla*_CMY-2_ found on different plasmid backbones (IncA/C (accession number: CP014658), IncK2 (accession number: KR905384), IncI1-pST2 (accession number: KT186369), IncI1-pST12a (accession number: NC_011077), IncI1-pST12b (accession number: CP012929)) compared to the genetic surrounding of *bla*_CMY-2_ in *S. enterica* serovar Newport (accession number: DQ164214). **b** Genetic surrounding of *bla*_CMY-2_ in eight *E. coli* isolates with chromosomally encoded *bla*_CMY-2_. Isolates with twofold encoded *bla*_CMY-2_ are indicated by an asterisk, the second copy was putatively chromosomally integrated. **c** Putative chromosomal integration of *bla*_CMY-2_ in 15 isolates compared to a previously published sequence (accession number FM246884). The presence of a *Shigella flexneri* 2a SRL pathogenicity island region downstream of truncated genes *blc* and *yggr* is shown. Isolates with two *bla*_CMY-2_ genes (additional putative chromosomal integration site) are indicated by an asterisk, respectively

All 72 IncK2 plasmids showed the previously described genetic environment of *bla*_CMY-2_, consisting of IS*Ecp1* upstream and *blc* and *sugE* downstream of *bla*_CMY-2_ (Fig. 2a) [18, 19]. However, in two isolates a sequence alteration was observed: Isolate no. 66–08 showed an insertion element (IS*Kpn26-like*) integrated between IS*Ecp1* and *bla*_CMY-2_. Isolate no. 35–16 had a truncated IS*Ecp1* element due to an IS*Kpn26* integration. Transconjugants harbouring both *bla*_CMY-2_-carrying plasmids were resistant to cefotaxime, ceftazidime and cefoxitin confirming the functionality of this beta-lactamase gene despite sequence alterations upstream of *bla*_CMY-2_.

Our genome data showed high nucleotide sequence identity (99.8–100%) when the shufflon region was excluded for 13 isolates (human *n* = 2; poultry *n* = 5; poultry meat *n* = 6) compared with plasmid pTMSA1088 (Genbank accession no: KR905386.1); plasmid sizes ranged from 79.3 kb to 80.6 kb. Insertion of an IS*2* element into different locations of the plasmid sequence was found in two of the 13 isolates. The majority of IncK2 plasmids (53/72 isolates) were identical to plasmid pDV45 (KR905384.1). This highly conserved plasmid sequence was found in isolates from human patients (*n* = 11), broiler chicken (*n* = 13), pigs (*n* = 3), broiler meat (*n* = 25) and turkey meat (*n* = 1). A nucleotide sequence identity of 98.6–100% was observed when the shufflon region and unique MGEs were excluded. In eleven of these isolates, the plasmids showed unique insertion sites of various MGEs (Additional file 6: Table S2**).** One isolate (no. 466–16, ST117, chicken) carried both, a pDV45-like plasmid and an IncI1 plasmid (type IncI1 pST12b, see below) carrying *bla*_CMY-2_.

In six isolates (all *E. coli*-ST429; chickens n = 3 and chicken meat n = 3) the IncK2 plasmid backbone was reconstructed, but showed an additional unique nucleotide sequence compared to plasmid pDV45 (Additional file 5: Figure S4). The plasmid sizes ranged after de novo assembly from 114.5–120 kb (confirmed by S1 nuclease PFGE for isolate no. 8–16) and varied due to inserted MGEs (Additional file 6: Table S2). The additional nucleotide sequence as compared to pDV45 included an undescribed Tn*As3*-like-element, interrupted by an IS*1326* element, a mercury resistance operon, genes encoding plasmid stability proteins (*stbAB*) and a colicin operon.

**Fig. 3:**
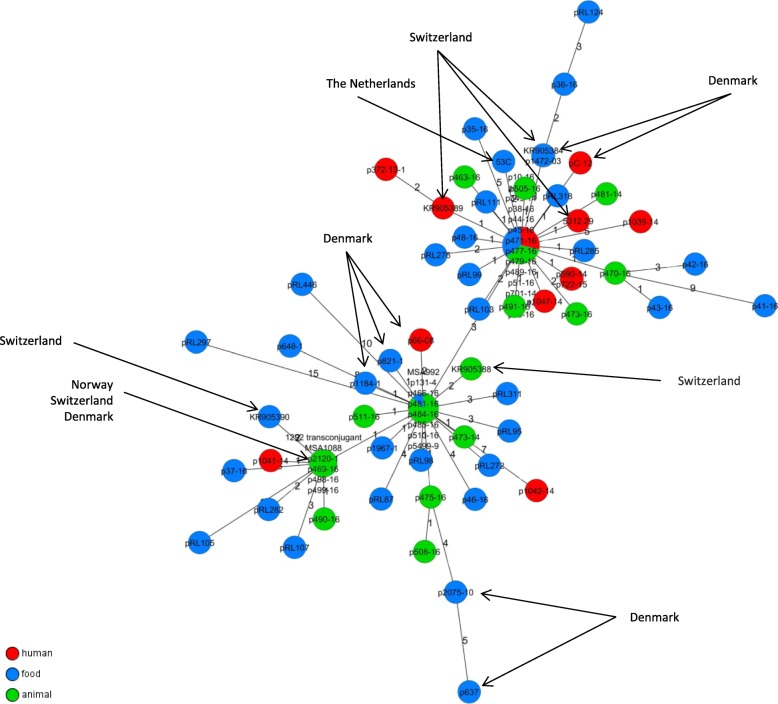
Minimum spanning tree of IncK2 plasmid sequences based on a maximum common genome approach using a gene-by-gene comparison in SeqSphere+ (v. 4.0.1, Ridom GmbH, Münster, Germany). We included 67 genes for this comparison. Previously published plasmids not from this study  (Germany) are indicated by arrows

The plasmid-wide maximum common-gene-approach by a gene-by-gene comparison was conducted for all IncK2 plasmids similar to pDV45 and pTMSA1088. Additionally available sequence data (from Genbank) of further IncK plasmids harbouring *bla*_CMY-2_ were included into this comparison. Results revealed a high level of similarity: The amount of differences varied between 0 and a maximum of 20 genes. Nevertheless the majority of investigated plasmid sequences exhibited variations in 0 up to 2 genes. Additional *bla*_CMY-2_-containing IncK2 plasmids sequences from previous studies conducted in Europe showed identical allele pattern to plasmid sequences from this study or differed by a maximum of 4 alleles in pairwise comparisons to plasmids from Germany (Fig. 3**).**

#### IncI1-Iα plasmids

Further replicon typing by the PlasmidFinder software revealed in 81 isolates the presence of an IncI1 sequence (Additional file 1: Table S1). This replicon sequence and *bla*_CMY-2_ were found on one contig in 32 isolates. For another 29 isolates, this linkage was confirmed by alignments to reference sequences of IncI1 plasmids. Thus, 61 isolates (human *n* = 17; chicken *n* = 20; pig *n* = 2; turkey *n* = 1; chicken meat *n* = 16; turkey meat *n* = 4; pork *n* = 1) carried *bla*_CMY-2_ on IncI1 plasmids. The existing pMLST scheme for IncI1 plasmids was used to classify these plasmids into different backbone lineages: pST12 (*n* = 39), pST2 (*n* = 12), pST43 (*n* = 2), pST23, pST27, pST26, pST36, pST55, pST240, pST241, and pST242 (each *n* = 1). Plasmids of pST12 presented two types of the *bla*_CMY-2_ integration into the plasmid backbone: IncI1 pST12a (*n* = 25) and IncI1 pST12b (*n* = 12). The plasmid backbone of four isolates, identified as pST12 with *bla*_CMY-2_, could not be determined.

IncI1 pST12a plasmids (chicken/broiler n = 12; human *n* = 5; pig *n* = 1; turkey n = 1; chicken meat *n* = 6) revealed a high similarity (98.5–99.8% pairwise identity) to plasmid pCVM29188_101 (Genbank accession no: NC_011077.1; 101 kb) from a *Salmonella enterica* isolate of poultry origin (USA, 2003) and only differed from that by absence of a 2462 bp IS*Ec22* in the pilus region. The direct integration site of *bla*_CMY-2_ and the flanking genes differed in comparison to the genetic environment of *bla*_CMY-2_ found in IncI1 pST12b plasmids similar to p12-4374_96 (Genbank accession no: CP012929.1) (Fig. 2a). Plasmid pCVM29188_101 contained a 3915 bp sized fragment (IS*Ecp1*- *bla*_CMY-2_ - *blc* - *sugE*) that was integrated into the *yagA* gene. In plasmid p12-4374_96 the *bla*_CMY-2_ fragment was 3123 bp in size and contained IS*Ecp1*- *bla*_CMY-2_ and a 65 bp truncated version of *blc.* The integration site of this *bla*_CMY-2_ fragment is located in the reading frame for a hypothetical/uncharacterized protein. In addition, insertions of MGEs were observed in eight isolates of the type IncI1 pST12a (Additional file 6: Table S2).

IncI1 pST12b plasmids (human *n* = 1; chickens *n* = 5; chicken meat *n* = 3; turkey meat *n* = 3) exhibited a high level of sequence identity (93.7–99.9% pairwise identity) with a previously described 96 kb plasmid (p12-4374_96: Genbank accession no: CP012929.1) from a human clinical *Salmonella* strain from Canada in 2012 [20]. Unique integration sites for MGEs were observed in IncI1 plasmids of five isolates (Additional file 6: Table S2).

The second most common IncI1-pST was pST2 (humans *n* = 3; pig *n* = 1; chicken *n* = 1; chicken meat *n* = 6; pork *n* = 1), and these plasmids showed high similarity (nucleotide sequence identity 96.7–99.9%) to plasmid pC-6 (Genbank accession no: KT186369.1) from a human urine sample (2009, Denmark). In nine of the twelve pST2 plasmids, unique integration sites of MGEs were observed (Additional file 6: Table S2). Interestingly, only in plasmids of *E. coli*-ST58 isolates (no. RL89, no. RL225, no. RL226 and no. 47–16) an insertion of IS*Ec12* into the reading frame of a hypothetical protein was found.

The plasmid backbones of IncI1 pST43 (*n* = 2), pST23, pST27, pST26, pST36, pST55, pST240, pST241, pST242 (each *n* = 1) were not reconstructed but the direct genetic environment of *bla*_CMY-2_ in these plasmids was identical to the IS*Ecp1*-*bla*_CMY-2_-*blc*-*sugE* structure (Fig. 2a).

#### IncA/C plasmids and chromosomal integration of *bla*_CMY-2_

Replicon sequence for IncA/C group and *bla*_CMY-2_ gene were found on the same de novo assembled contig of four isolates (humans *n* = 2; turkey meat *n* = 1; pork *n* = 1). The contigs and aligned reads showed high resemblance (up to 98.9% nucleotide sequence identity at 90.9% coverage) to the 160 kb-IncA/C plasmid pSAN1–1736 from *Salmonella enterica* from bovine skin (GenBank accession no. CP014658.1). S1-PFGE of a transconjugant of isolate no.761–14 confirmed the presence of a *bla*_CMY-2_-carrying IncA/C plasmid with an estimated size of 170 kb. The *bla*_CMY-2_ gene was in all four isolates flanked by IS*Ecp1* upstream and *blc* and *sugE* downstream (Fig. 2a).

For ten (6.1%) of the 164 CMY-2-producing *E. coli* isolates (humans *n* = 7; turkeys *n* = 2; pig *n* = 1) the *bla*_CMY-2_ gene was found to be integrated into chromosomal genes. As expected *bla*_CMY-2_ transfer by broth mate conjugation was unsuccessful for these isolates. The integration of *bla*_CMY-2_ appeared for eight of ten isolates at a unique site and two isolates (no. 90–16, 216–16) at the same site in the chromosome (Fig. 2b). Four of these eight isolates (no. 501–16, 502–16, 509–16 and 739–12) showed a twice as high coverage of the IS*Ecp1-bla*_CMY-2_ sequence compared to adjacent sequence regions, indicating a second integration site of *bla*_CMY-2_. Detailed analysis of the contigs and reads revealed for these four isolates the same additional *bla*_CMY-2_-containing genetic environment (Fig. 2c): IS*4* was adjacent to the IS*Ecp1*-*bla*_CMY-2_ sequence; downstream of *bla*_CMY-2_ a truncated *blc* gene (*Δblc*) was observed, which was followed by a truncated *yggR* gene (*ΔyggR*) and a *Shigella flexneri* 2a SRL pathogenicity island-like region (95% identity). This conserved structure was identical to the previously reported genetic environment TN44889 of *bla*_CMY-2_ (GenBank accession no. FM246884) and described to be chromosomally integrated by Fang et al. [18, 21]. However, a direct chromosomal integration of the additional *bla*_CMY-2_-containing genetic environment could not be confirmed in the four isolates due to the above mentioned mobile genetic elements being adjacent to this sequence.

In ten further isolates (humans *n* = 5; turkey *n* = 1; pigs *n* = 2; cattle *n* = 1; turkey meat *n* = 1) we found only the putative, chromosomally integrated *bla*_CMY-2_-environment described by Fang et al. [21]; plasmid replicons were not identified. Likewise no transfer of *bla*_CMY-2_ by broth mate conjugation appeared. In addition, these isolates showed a nucleotide sequence downstream of TN44889 identical to the draft genome of *E. coli* Co6114 (GenBank accession no. CP016034.1) (Fig. 2c) [22].

## Discussion

In the present study, we compared core genome sequences and plasmid sequences of 164 *bla*_CMY-2_-harbouring *E. coli* isolates from meat products (*n* = 63), livestock animals (*n* = 55), and human patients (*n* = 46) to assess national or international transmission of CMY-2-producing strains or *bla*_CMY-2_-carrying plasmids. Until today, only a few NGS-based transmission analyses of CMY-2-producing *E. coli* across sectors have been published despite knowing prevalence rates of more than 20% in poultry and poultry meat [7, 9, 10]. In human patients in Europe, ca. 1% of the 3rd generation cephalosporin-resistant *E. coli* produce CMY-2 [5, 23, 24]. However, in recent studies from Asia much higher rates and an increasing trend among 3rd-generation cephalosporin-resistant *E. coli* isolates have been reported [25, 26]. Moreover, studies from Denmark, Norway and the Netherlands suggest that *bla*_CMY-2_ transmission along the food chain is probable due to either horizontal transfer events or clonal spread [7, 9, 10, 27]. Interestingly, whole genome analyses of ESBL-producing *E. coli*-ST410 that were found in various sources (human patients, healthy humans, livestock animals, pets and wastewater samples) indicated a high stability of this clonal lineage and a transfer between different reservoirs [27, 28].

### Distribution and proportion of clonal lineages – MLST and cgMLST

In this study MLST analysis was done first to provide an overview about the present CMY-2-*E. coli* population structure, and to enable the comparison with results of previous studies. The 164 CMY-2-producing isolates could be assigned to 59 different STs, and the isolates from human patients showed a higher ST variety than the isolates from livestock animals or meat products (Table 1). The more frequently observed STs, e.g. ST38 (*n* = 19), ST131 (*n* = 14), ST117 (*n* = 14), and ST69 (*n* = 8), were also found to be prevalent in studies on ESBL-producing *E. coli* from retail poultry meat, livestock animals and human patients indicating a successful spread of clonal lineages independent from distinct resistance genes [3, 29]. These STs were previously described in other European countries (Norway, Denmark, Sweden, Switzerland and Netherlands) demonstrating the high variability of *bla*_CMY-2_-harbouring *E. coli* lineages in all sources (human/livestock/food) [7–9, 30]. Interestingly, we found CMY-2-producing *E. coli*-ST131 in equal proportions in human patients (*n* = 6) and chickens (*n* = 6) and in some meat products (*n* = 2). In contrast, ESBL-producing *E. coli*-ST131 isolates have been found in previous studies only occasionally in samples of animal origin [7, 31]. On the other hand, CMY-2-producing *E. coli*-ST38 isolates were found exclusively in poultry and poultry meat, whereas this ST has been rarely described in ESBL-producing isolates from humans [11].

A putative clonal transfer along the food chain cannot be drawn from results of typing methods with a comparably low discrimination such as MLST, even in combination with additional data, such as resistance pattern, plasmid replicon type and beta-lactamase gene presence [10]. Hence, we conducted a genome-wide gene-by-gene comparison. The clustering of isolates in our cgMLST scheme was in good concordance with their respective ST groups, but not always identical (e.g. ST10, Fig. 1). ST10 *E. coli* isolates were noticeable less genetically related than isolates of other STs. This high diversity within ST10 is known and can be explained by the chosen most common MLST scheme (“Warwick scheme”) and the corresponding housekeeping genes, which are not sufficiently discriminative for ST10 compared to the housekeeping genes from the two other available MLST schemes (“Pasteur and Michigan scheme”) [32–35]. This highlights the importance of using typing techniques with a higher discrimination than MLST to avoid wrong assumptions about genetic relatedness or transmission events. Nevertheless, we used the ST nomenclature to enable an international comparison and a differentiation of isolates that grouped together in our cgMLST scheme.

Within a ST, isolates from human patients mostly showed a larger distance to isolates from livestock or food, but all seemed to have a common ancestor. Sporadic clonal transmission events between different sources (human, livestock, food) in the past followed by parallel independent micro-evolution within the different sources might explain this differentiation between isolates from human and livestock/food isolates [3]. In fact, only within ST1463 and ST131 a very high similarity of isolates from human and animal/food origin was detected in cgMLST and in subsequently performed SNP-based analysis (Additional file 3: Figure S2b). Since these isolates were either from the same geographical region (federal state) or from the same period of time (6–12 month distance), a clonal transmission seems likely. Recently, a research group from Norway reported nearly identical strains of CMY-2-producing *E. coli*-ST38 (SNP differences 1–13) from chickens and from human patients indicating a clonal transmission as well [9].

Furthermore, we found a close relationship of isolates from poultry and poultry meat products, especially for *E. coli* isolates of ST117, ST3778 and ST429 (Additional file 3: Figure S2a,d). The subsequently performed SNP analysis of isolates of ST117 and ST3778 revealed high similarities with 0 to 40 SNPs differences between the isolates. The identified numbers of discriminating SNPs are equal or marginally higher than SNP differences reported previously from isolates derived from clonal outbreaks [10, 36]. In previous studies, epidemiologically linked isolates exhibited SNPs ranging from ≤6, (isolates of a big German EHEC outbreak) ≤ 4 (epidemiologically linked cases of *E. coli* O157 from Scotland) or ≤ 23 (*E. coli* O157 pork-associated outbreak in Alberta, Canada) [36–39].

In a study by Mellmann et al. a threshold of < 11 alleles difference in a pairwise comparison of isolates was used to identify nosocomial transmissions of *E. coli* in an ad-hoc cgMLST analysis (based on 2325 alleles) [40]. Applying this threshold to the analyzed ST429 isolates with *bla*_CMY-2_, the relationship of nearly all isolates would indicate clonal transmission. Taken into consideration that plasmid analyses of these isolates also showed highly similar plasmid sequences to the p486–16 plasmid sequence, a clonal transmission of an *E. coli* ST429 strain carrying a *bla*_CMY-2_-carrying IncK2 plasmid seems likely. This supports the hypothesis of clonal transmission of *bla*_CMY-2_ along the poultry production chain and across sectors. The study design did not enable a direct observation of individual animals from breeding to slaughter, whereas this argument remains speculative to a certain extent. However, veterinary studies in other countries support this hypothesis. In the Danish conventional broiler production, the spread of ESBL-producing *E. coli* clones and ESBL gene-carrying plasmids were observed in imported broiler parent flocks, despite cephalosporins have been never approved for use in poultry [41]. Furthermore, a relatively stable colonization with CMY-2-producing *E. coli* was observed in a Norwegian study through the whole broiler production chain from grandparent animals to retail meat [23]. Cross-contaminations during the successive fattening of several herds in the same stable due to insufficient cleaning and cross-contaminations in big slaughterhouses, where animals from many farms were processed, facilitate the spread of distinct clones [42].

### Resistance and virulence

Studies on ESBL-producing *E. coli* of human origin reported rates of 60–70% fluoroquinolone resistance [11, 43]. Our data showed that 43.5% of the *bla*_CMY-2_-harbouring isolates from human origin were resistant to ciprofloxacin. Differences in the observed fluoroquinolone resistance rates could be explained by the occurrence of different proportions of *E. coli*-ST131 in ESBL-*E. coli* compared to CMY-2-*E. coli* [12]. The prevalence of *E. coli*-ST131 is observed up to 70% among ESBL-positive *E. coli* associated with nosocomial or ambulant urinary tract infections [12]. Further, CMY-2-producing *E. coli*-ST131 isolates belong mainly to the fluoroquinolone-susceptible ST131 clade B (*fimH*22; see also next chapter) and not to clade C (*fimH*30); the latter comprises mostly strains resistant to fluoroquinolones [12]. However, rates of fluoroquinolone-resistant CMY-2-*E. coli* from livestock animals or meat products were comparable to the rates of resistance to nalidixic acid and ciprofloxacin among *E. coli* from broilers in Sweden, but noticeable lower than in human *E. coli* isolates [44].

The recently described colistin resistance gene *mcr-1* was observed additionally in twelve CMY-2-positive *E. coli* strains from this study in chicken (*n* = 3), pig (*n* = 5), turkey (*n* = 1) and chicken meat products (*n* = 3). *Mcr-1* in Germany is found predominantly in the poultry production chain [45]. The higher number of findings of *mcr-1* in isolates from pigs compared to isolates from chicken in the present study is most likely biased by the isolate selection process for this study.

In addition, in one isolate from a diseased pig (no. 6–16, ST10, serotype O141:H49) analysis exhibited the presence of *stx-2*. Shiga toxin-production has been rarely described in ESBL/AmpC-producing *E. coli* so far [46, 47].

### CMY-2-producing *E. coli*-ST131

We identified a substantial amount of *E. coli*-ST131 isolates (human patients 6/46, 13.0%; poultry 6/45, 13.3%; food 2/63, 3.2%), collected from 2010 till 2016. Regarding ESBL-producing *E. coli*, ST131 is the most frequent ST in humans but occurs only sporadically in pets, livestock or wild animals (0–1.8% of 3rd generation cephalosporin-resistant *E. coli*) [3, 11, 12]. All but one CMY-2-producing *E. coli*-ST131 isolates from the present study harboured *fimH*22 and clustered in clade B. The single non-*fimH*22 isolate (no. 177–10) belonged to the successful ST131 subclone *H*30-Rx and carried the putative chromosomally encoded ESBL gene *bla*_CTX-M-15_. The comparison of 15 published whole genome sequences of *E. coli-*ST131 isolated between 2008 and 2017 with *bla*_CMY-2_, using the ad-hoc cgMLST approach, showed that nine isolates from other countries likewise contained *fimH22* and differed in 3 to 113 target genes from CMY-2-producing *E. coli-*ST131 isolates from this study (Additional file 4: Figure S3). The affiliation of our CMY-2-producing *E. coli*-ST131 into clade B (*fimH22*) in contrast to the ESBL-producing *E. coli-*ST131 of clade C (ST131-O25b *fimH*30-R/*fimH*30-Rx) suggests a CMY-2-producing *E. coli*-ST131 population with an independent evolution in different reservoirs. ST131-O25b with ESBL-production often occurs in human patients and isolates might be adapted to colonization and infection in the human bladder [48]. In contrast, CMY-2-producing *E. coli*-ST131 isolates were described rarely in humans [31, 49, 50] and recent studies from Germany, the Netherlands and Denmark reported only a few isolates in poultry [7, 17]. The much higher proportion of ST131 in our CMY-2-producing *E. coli* collection remains unclear; we did not recognize any obvious bias in the sampling strategy of the underlying studies for the isolate collection which might have influenced enrichment for ST131 clade B (*fimH22*) isolates in our sample.

### Plasmid structures

The integration of the *bla*_CMY-2_ gene occurred in a narrower spectrum of genetic structures and plasmid backbones in relation to the comparably high variety of clonal lineages. *bla*_CMY-2_ has been frequently reported on IncI1, IncK and IncA/C plasmids in *E. coli* and sporadically on IncF, IncX, IncI2 or IncHI2 plasmids [21, 51–53]. However, in our collection we only identified IncI1, IncK and IncA/C plasmids carrying *bla*_CMY-2_. The most prevalent plasmid types were of incompatibility groups IncK2 (*n* = 76) and IncI1 (*n* = 61). Previous studies reported that dissemination of *bla*_CMY-2_ in *E. coli* is mainly driven by plasmids of these two Inc groups within Europe [7–9, 54]. In contrast, IncA/C plasmids seem to play a more prominent role in the spread of *bla*_CMY-2_ in North America [18, 55–57]. In a Dutch study all *bla*_CMY-2_-harbouring plasmids belonged to IncA/C indicating the importance of clonal spread in isolated populations such as broiler stocks [58].

Our analysis revealed that all IncK plasmids belonged to the recently described IncK2 type [8]. Sequence identity among these plasmids was high and the most frequent plasmid backbone structures were similar (98.6–100% identity) to two previously published plasmids pTMSA1088 (*n* = 13) (KR905386.1) and pDV45 (*n* = 54) (KR905384.1) [8]. Plasmids similar to pTMSA1088 were found in isolates of ST38 (*n* = 11), ST10 (*n* = 1) and ST117 (*n* = 1). This specific ST38/pTMSA1088-like combination has been reported before in broiler meat production of several countries (Netherlands, Denmark, Norway, Sweden) and raised the discussion if a highly conserved IncK2 plasmid carrying *bla*_CMY-2_ is genetically linked to ST38 [7–9]. In a direct gene-by-gene comparison of all pTMSA1088-like and pDV45-like plasmids from the present study with *bla*_CMY-2_ plasmids from the Netherlands, Denmark, Norway and Switzerland, we confirmed the expected high relatedness. The low number of variations observed in the genes of these plasmids indicates a horizontal transmission, and for the ST38/pTMSA1088-like combination a dissemination of the plasmids linked to a clonal transmission across the European broiler production is highly probable (Fig. 3) [7, 8, 10, 59]. Moreover, a modification of the plasmid backbone structure in comparison to plasmid pDV45 was observed in six ST429 isolates (Additional file 5: Figure S4**).** The occurrence of this backbone structure only in ST429 suggests a clonal transmission linked to the plasmid. Identical IS insertions in the plasmids and SNP-based comparisons of the core genomes (Additional file 3: Figure S2d) support this suggestion. The modified plasmid backbone structure contained additional genes encoding a plasmid segregation system (*stbAB*) which enables a stable vertical transmission during cell division as well as horizontal transmission (conjugation), and thus it promotes efficient *bla*_CMY-2_ plasmid propagation [60].

In contrast to the IncK2 plasmids, we detected more diverse IncI1 plasmid structures. IncI1 plasmids carrying *bla*_CMY-2_ were reported worldwide and differ considerably in their plasmid backbone structures [7, 18, 61, 62]. In the present study most IncI1 plasmids could be assigned to pST12 (39/61) and pST2 (12/61). The majorities of IncI1 plasmids of each respective pSTs were highly similar in their conserved backbone structures and differed only in a few SNPs or an MGE insertion. Among IncI1-pST12 plasmids two different types (IncI1 pST12a and IncI1 pST12b) were identified differing in the direct genetic surrounding of *bla*_CMY-2_ and an additional plasmid addiction system. These differences in the integration site of *bla*_CMY-2_ indicate two independent mobilization events into highly similar plasmid backbones.

In general, the distribution of *bla*_CMY-2-_carrying IncK2 and IncI1 plasmids was heterogeneous among isolates from human patients, livestock animals and food as well as in various *E. coli*-STs (Fig. 1) which supports the hypothesis that the horizontal transfer via plasmids plays the major role for transmission of *bla*_CMY-2_ between the reservoirs. Given the capability of these plasmids to transfer themselves within *E. coli* and into other *Enterobacteriaceae* genera, the ingested *bla*_CMY-2_-carrying strains from meat products might transfer their resistance gene to the human host-adapted strains [63].

### *bla*_CMY-2_ in the chromosome

In 20 isolates, mainly from human patients (12/20), the localization of *bla*_CMY-2_ on the chromosome was either confirmed or suspected to be chromosomal. While for ten isolates *bla*_CMY-2_ was found to be located on the chromosome by sequence and read analysis, for further ten isolates the localization of *bla*_CMY-2_ on the chromosome was suspected due to the identity to a previously described *bla*_CMY-2_ structure: A recent publication from China reports the integration of this *bla*_CMY-2_-containing fragment TN44889 into the chromosome of *E. coli* strains (9/469) isolated from companion and livestock animals [21]. It is known that *bla*_CMY-2_ can integrate into the chromosome of *Proteus mirabilis* or *S. enterica* but only in few studies isolates with *bla*_CMY-2_ integrated into the chromosome of *E. coli* were identified [64, 65]. The variety of confirmed and presumable chromosomal integration sites and the differences in the *bla*_CMY-2_genetic environments in general point towards independent events of chromosomal integration of this resistance gene. However, the high similarity (2 to 3 SNP-differences) of three ST1196 isolates from livestock animals from our collection (Additional file 3: Figure S2c) with an identical insertion site of IS*Apl1* suggested propagation by clonal transmission in this specific setting. The reason for the comparable high number of isolates from humans (*n* = 12) with chromosomal *bla*_CMY-2_ integration sites in this study remains unknown.

### Limitations

The study has several limitations to be considered. First, the sampling of the 164 CMY-2-producing *E. coli* isolates was conducted over a period of 5 years in the scope of different studies (human patients, livestock animals, food) of the national research project RESET. Regarding studies on livestock animals, we selected only one isolate from each farm or isolates from the same farm with different properties, e.g. different phylogenetic groups, to achieve a preferably heterogeneous sample of the distribution of *bla*_CMY-2_-carrying isolates in German farms. Therefore, the selection of the isolates, especially from broiler production, is not completely randomized but as diverse as possible. Second, the long sampling period of five years was necessary due to the comparably low prevalence of CMY-2-producing *E. coli* among 3rd-generation cephalosporin-resistant *E. coli* from human infections and colonizations. Third, putative links between isolates from animals, food and humans could not be further elucidated due to limited clinical data of patients and missing information on origin and transport ways of livestock animals and meat products.

## Conclusions

The whole genome sequence analysis of 164 CMY-2-producing *E. coli* isolated from human patients, livestock animals and meat products in Germany revealed a high diversity of STs across all sources; most frequent types were ST38, ST131 and ST117. The CMY-2-producing ST131 isolates from human patients and livestock animals belonged to another sub-lineage (clade B; *fimH22*) than the worldwide prevalent multidrug-resistant and ESBL-producing ST131-O25b lineage (clade C; *fimH*30) known from humans. Our data suggest that clonal transmission of *bla*_CMY-2_ is a rare event while the horizontal transfer of temporally stable *bla*_CMY-2_-carrying IncK2 and IncI1 plasmids is more likely the dominant way of transmission between humans and animals. At present, CMY-2-carrying *E. coli* are rarely detected from human infections in Germany and Central Europe. However, the large reservoir of *bla*_CMY-2_ -carrying plasmids in livestock animals, especially poultry, poses a serious future risk of a more pronounced potential of CMY-2-producing *E. coli* isolates causing infections in humans and animals.

## Methods

### Bacterial isolates

We included 164 CMY-2-producing *E. coli* from Germany collected 2008–2016. The majority of isolates (*n* = 149) was collected and isolated between 2011 and 2014 in the scope of the national research consortium “RESET” (www.reset-verbund.de) by different project partners: Robert Koch Institute (RKI), German Federal Institute for Risk Assessment (BfR), Friedrich-Loeffler-Institut (FLI) and Freie Universität Berlin (FU). No concrete sampling plan for the collection of the isolates was developed; rather all isolates were collected in the scope of different studies including isolates from healthy livestock animals, diseased livestock animals, food samples and human patients, and represent therefore a random collection of CMY-2-producing *E. coli* isolates from Germany (Additional file 1: Table S1). To enable a higher comparability of studies on different animal species, the RESET consortium harmonized protocols for sampling and basic phenotypical and genotypical analyses were developed in advance. FU provided 50 CMY-2-producing *E. coli* isolates from chicken (*n* = 9 in 2011 and *n* = 28 in 2012), turkey (*n* = 1 in 2012 and *n* = 3 in 2014), pig (*n* = 6 in 2011 and *n* = 2 in 2012) and cattle (*n* = 1 in 2012) for WGS analysis. These isolates were collected in longitudinal- and cross-sectional studies in different livestock farms during the years 2011–2014 [66–70]; only isolates from unique production sites were included. In addition, BfR provided 63 CMY-2-producing *E. coli* isolates that were identified from 2500 food samples of different origins and matrices, collected and investigated by different German state laboratories, in the scope of a cross-sectional study on ESBL-*E. coli* in foodstuff in Germany (unpublished data). In brief, the 63 isolates were from chicken meat samples (*n* = 53), turkey meat samples (*n* = 7) and pig meat samples (*n* = 3), and were isolated between 2011 and 2013. Furthermore, FLI identified five CMY-2-producing *E. coli* isolates from diseased livestock animal (chicken *n* = 3, pig *n* = 1 and cattle *n* = 1) that were included in the present study [71]. These five isolates were detected between 2008 and 2015 within the GE*RM*-Vet program, a monitoring program collecting data of resistance of pathogenic bacteria and including only isolates from clinically diseased, non antibiotically pretreated animals. Finally, CMY-2-producing *E. coli* from human clinical samples (*n* = 46) were collected by RKI between 2008 and 2016. These isolates were obtained from different studies [5, 11] or were sent from German laboratories to RKI for confirmatory diagnostics (Additional file 1: Table S1).

### Antimicrobial susceptibility testing and resistance gene screening

Susceptibilities to ampicillin, cefotaxime, ceftazidime, cefoxitin, ciprofloxacin, imipenem and meropenem were tested for all *E. coli* isolates by disk diffusion (Oxoid Ltd., Basingstoke, United Kingdom) according to the manufacturer’s instructions, and interpretation was done according to EUCAST criteria (http://www.eucast.org/clinical_breakpoints; v7.1). The phylogenetic group of the *E. coli* isolates was determined by a PCR-based assay [72]. The presence of β-lactamase genes (*bla*_TEM_, *bla*_SHV_, *bla*_CTX-M-group-1-2-9_, and *bla*_CMY_) was confirmed by PCR and sequencing as previously described [73, 74]; only CMY-2 positive isolates were included in the present study.

### *bla*_CMY-2_ transferability and plasmid typing

A broth mate conjugation assay was performed for 60 randomly selected isolates using the sodium azide-resistant *E. coli* K12 J53 recipient strain [75]. Transconjugants were selected on Lysogeny-Broth (LB) agar supplemented with 200 mg/L sodium azide and 100 mg/L ampicillin. Selected transconjugants were tested for the presence of *bla*_CMY-2_ by PCR as previously described, and the plasmid sizes were determined using S1 nuclease-restriction followed by pulsed-field gel electrophoresis (PFGE) [73, 76]. Plasmidic DNA was extracted by the Qiagen Plasmid mini Kit (Hilden, Germany) and followed by PCR-based replicon typing (PBRT-Kit, Diatheva, Italy).

### Whole genome sequencing and sequence reconstruction

All 164 *bla*_CMY-2_-containing *E. coli* isolates were sequenced using an Illumina MiSeq platform (Illumina, San Diego, U.S.A). Isolate preparation, DNA extraction and sequencing were carried out by RKI, BfR and LGC Genomics GmbH (Berlin, Germany). At the RKI genomic DNA of 58 *E. coli* isolates was extracted using the DNA Blood & Tissue Kit according to the manufacturer’s instruction (Qiagen, Hilden, Germany), followed by library preparation, using Nextera XT library (Illumina, USA), and sequenced on an Illumina MiSeq platform using the MiSeq v3 reagent kit (Illumina, USA) with 2 × 300 bp paired-end reads. Furthermore, 62 *E. coli* isolates were sent to the company LGC Genomics that performed DNA extraction and sequencing, using the Illumina MiSeq platform MiSeq v3 reagent kit (Illumina, USA) with 2 × 300 bp paired-end reads. At the BfR, extraction of genomic DNA of 44 isolates was carried out using PureLink® genomic DNA Kit from Invitrogen™ (by ThermoFisher Scientific).

The following RKI internal procedure was used: The resulting raw reads were processed by Trimmomatic (v. 0.0.9; default parameters except maxinfo 15:0.5) and assembled by A5-miseq (v. 0.0.9 beta; default parameters) [77, 78]. The quality of the read data was assessed by using the PHRED scores. Raw sequence data had an average PHRED score above 30. The average read quality was increased by trimming to a PHRED score of above 35.

### Phylogenetic analyses

All contigs were submitted to the CGE Finder Series (Centre for Genomic Epidemiology, Technical University of Denmark (DTU), https://cge.cbs.dtu.dk/services/). Different analysis tools (MLST 1.8, pMLST 1.4, PlasmidFinder 1.3, ResFinder 2.1, VirulenceFinder 1.5) were applied to extract the multilocus sequence type (ST) according to Wirth et al., plasmid multilocus sequence type (pST), plasmid replicon types and information on genes mediating resistance to β-lactams and fluoroquinolones and distinct virulence genes (*stx1/2*), respectively [32, 79–83].

For deeper phylogenetic analysis (i) a gene-based comparison approach and (ii) a single nucleotide polymorphisms (SNP)-based mapping analysis were performed. For this purpose, an ad-hoc core genome multilocus sequence typing scheme (cgMLST) was created using SeqSphere+ (v. 4.0.1, Ridom GmbH, Münster, Germany) as described before [84, 85]. *E. coli* O157:H7 str. Sakai (GenBank accession no. NC_002695.1) was used as reference genome and further 43 *E. coli* genomes from the National Centre for Biotechnology Information (NCBI) were selected as query genomes. Open reading frames (ORFs) were predicted and extracted by using the cgMLST Target Definer v1.4 of SeqSphere+, resulting in 2547 shared genes among these isolates, which were defined as core genome (Additional file 7: Table S3). Obtained cgMLST target gene variants were used to visualize the phylogenetic distance by calculating a Neighbour-Joining tree (parameters were: pairwise ignoring missing values; % columns difference) based on the distance matrix of the core genome differences. The tree was visualized using iTOL (v. 3.5.4) (http://itol.embl.de/) [86].

Isolates which presented a high relationship in the cgMLST scheme were further investigated by single nucleotide polymorphisms (SNP)-based mapping analysis. A suitable reference was identified using refRank as described previously (https://gitlab.com/s.fuchs/refRank) [87]. Paired-end reads were mapped to the identified best reference genome for respective selected isolates (ST131: NZ_CP019008.1; ST117/ST3778: NZ_CP019903.1; ST1196: NC_020518.1; ST429: NC_013654.1) by BWA-SW (v. 0.7.15-r1140; default parameters) [88]. Variant calling was performed using VarScan (parameter: min-coverage 10; min-reads2 6; min-avg-qual 20; min-var-freq 0.8; min-var-for-hom 0.75; *p*-value 0.01; strand-filter 0) [89]. SNPs were filtered using SNPfilter (https://gitlab.com/s.fuchs/snpfilter) [87]. Maximum likelihood trees were calculated using RAxML with a GTR GAMMA nucleotide model (rapid hill-climbing, using 100 starting trees) [90]. Phylogenetic trees were visualized by iTOL (v. 3.5.4).

All *E. coli* isolates of ST131 were analysed for the presence of *fimH* gene by aligning to a *fimH* database using SeqSphere+ [14, 91].

### Plasmid analysis

Regarding the identification of the *bla*_CMY-2_-harbouring contigs, all contigs were aligned using Geneious (v. 10.0.5, Biomatters Ltd., Auckland, New Zealand) to the *bla*_CMY-2_ gene (GenBank accession no. X91840.1). Contigs containing *bla*_CMY-2_ were hereafter aligned to several fully sequenced *bla*_CMY-2_-carrying plasmids of different Incompatibility groups (Inc) from GenBank using Geneious to identify highest resemblance to a reference plasmid: IncK2: pDV45 (GenBank accession no. KR905384.1), pTMSA1088 (GenBank accession no. KR905386.1); IncI1: p12-4374_96 (GenBank accession no. CP012929.1), pC-6 (GenBank accession no. KT186369.1), pCVM29188_101 (GenBank accession no. NC_011077.1); IncA/C: pSAN1–1736 (GenBank accession no. CP014658.1).

All contigs of a respective isolate were aligned to the identified best fitting reference plasmid. Additionally, read alignment of trimmed reads (Trimmomatic v. 0.0.9; default parameters except maxinfo 15:0.5) to the selected reference plasmid using the Geneious (v. 10.0.5) mapper (medium-low sensitivity, no iterations) was performed. The results obtained were then compared by aligning with the respective reference plasmids and examined for MGEs, using the ISfinder (https://www-is.biotoul.fr/index.php) [92].

To compare IncK plasmids from this study with available IncK plasmids carrying *bla*_CMY-2_ from nucleotide sequence databases, a maximum common gene approach was conducted. For that purpose ORFs from the *bla*_CMY-2_-carrying IncK2 plasmid pDV45 were predicted and extracted by using the cgMLST Target Definer v1.4 of SeqSphere+, resulting in 89 genes, of which 67 were found in all the plasmids to be compared. Target gene distance was visualized by a minimum spanning tree (based on 67 targets, pairwise ignoring missing values).

### Nucleotide sequence accession numbers

Sequence data were submitted to the European Nucleotide Archive (http://www.ebi.ac.uk/ena) under the study accession number PRJEB23663.

## Additional files

Additional file 1: **Table S1.** Characteristics of 164 CMY-2 -producing *Escherichia coli* isolates from different sources, 2008–2013, Germany (selection for whole genome sequencing). (XLSX 26 kb)

Additional file 2: **Figure S1.** Neighbour-joining tree of ST429 *E. coli* isolates based on an ad-hoc cgMLST including 2547 alleles. The tree was built with SeqSphere+. Included isolate sequences originated from this study and were obtained as contigs assembled by http://enterobase.warwick.ac.uk/ (ERR2091318, ERR209121, ERR2091324, ERR2091328, ERR2091334, ERR2091342, ERR2091342, ERR2091348, ERR2091349, ERR2091357, ERR2091358, ERR2091419, ERR2091421, DRR102690, SRR3050857, SRR3098809, SRR3987496, ERR1619552, ERR1622238, ERR1622239, ERR1622406, ERR1622406, ERR1622407, ERR1595423, ERR1543414, ERR277049, ERR1415546, ERR1163310, ERR435146, SRR2000414). All isolates were investigated for the presence of *bla*_CMY-2_ and IncK2 RNAI sequence. Positive isolates are marked by red border. All *bla*_CMY-2_ carrying isolates exhibited a p486–16-like IncK2 plasmid sequence. The allele distance between two *bla*_CMY-2_ -carrying isolates is shown. (PDF 580 kb)

Additional file 3: **Figure S2.** SNP-based maximum-likelihood-trees of isolates of selected sequence types. a: Maximum-likelihood-tree of ST117 and ST3778 isolates. SNPfilter (d = 0, reference: NZ_CP019903.1 *E. coli* strain MDR_56) based tree, calculated with RAxML, GTR Gamma and rapid hill-climbing and 100 starting trees. b: Maximum-likelihood-tree of ST131 O25b:H4 fimH22 isolates. SNPfilter (d = 0, reference: NZ_CP019008.1 *E. coli* strain Ecol_AZ159) based tree, calculated with RAxML, GTR Gamma and rapid hill-climbing and 100 starting trees. c: Maximum-likelihood-tree of ST1196 isolates. SNPfilter (d = 0, reference: NC_020518.1 *E. coli* str. K-12 substr. MDS42) based tree, calculated with RAxML, GTR Gamma and rapid hill-climbing and 100 starting trees. d: Maximum-likelihood-tree of ST429 isolates. SNPfilter (d = 0, reference: NC_013654.1 *E. coli* strain SE15) based tree, calculated with RAxML, GTR Gamma and rapid hill-climbing and 100 starting trees. (PDF 174 kb)

Additional file 4: **Figure S3.** Minimum spanning tree of *E. coli* ST131 isolates from this study and INSDC and Enterobase based on an ad-hoc cgMLST including 2547 alleles. All *fimH* alleles and *bla*_CMY_ and *bla*_CTX-M_ genes are color-coded. Isolates from this study are marked with a red ring. (PDF 971 kb)

Additional file 5: **Figure S4.** Comparison of plasmid sequence p486–16 with other IncK2 plasmids created with EasyFig v.2.2.2 (http://mjsull.github.io/Easyfig/). Used reference plasmid sequence were pTMSA1088 (Genbank: KR905386.1), pDV45 (KR905384.1). (PDF 38 kb)

Additional file 6: **Table S2.** Unique integration sites for MGEs in IncK and IncI plasmids of the present collection of 164 CMY-2 producing *E. coli* isolates of various origins. (XLSX 15 kb)

Additional file 7: **Table S3.** List of chromosomal core genome genes used. (XLSX 136 kb)

## Abbreviations

BfR: German Federal Institute for Risk Assessment
CGE: Centre for Genomic Epidemiology
cgMLST: core-genome multilocus sequence typing
*E. coli*: *Escherichia coli*
ESBL: extended-spectrum β-lactamases
EUCAST: European Committee on Antimicrobial Susceptibility Testing
FLI: Friedrich-Loeffler-Institut
FU: Freie Universität Berlin
Inc.: Incompatibility groups
INSDC: International Nucleotide Sequence Database Collaboration
IS: Insertion sequence
LB: Lysogeny-Broth
MGE: Mobile genetic element
MIC: Minimum inhibitory concentration
MLST: Multilocus sequence typing
NCBI: National Centre for Biotechnology Information
NGS: Next-generation sequencing
ORF: Open reading frames
pMLST: Plasmid- Multilocus sequence typing
PBRT: PCR-based replicon typing
pST: Plasmid sequence type
RKI: Robert Koch Institute
SNP: single nucleotide polymorphism
ST: Sequence type
WGS: Whole genome sequencing
