# Supplementary figures and images for: Whole genome analyses of CMY-2-producing Escherichia coli isolates from humans, animals and food in Germany
Source: BMC Genomics. 2018 Aug 9;19:601. doi: 10.1186/s12864-018-4976-3 (PMC6085623; doi:10.1186/s12864-018-4976-3)

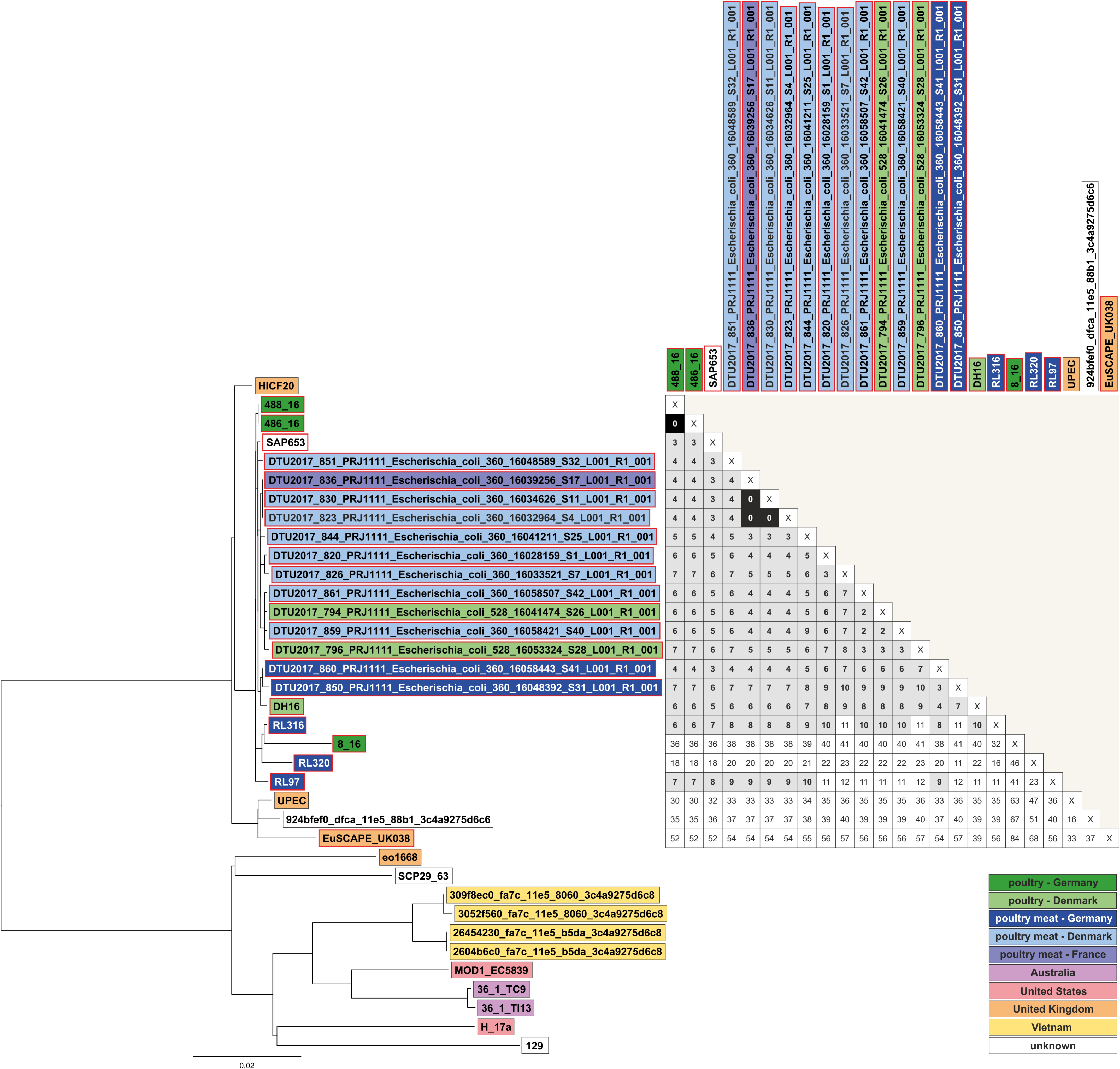

Supplement: Supplementary file 2 — Figure S1. Neighbour-joining tree of ST429 E. coli isolates based on an ad-hoc cgMLST including 2547 alleles. The tree was built with SeqSphere+. Included isolate sequences originated from this study and were obtained as contigs assembled by http://enterobase.warwick.ac.uk/ (ERR2091318, ERR209121, ERR2091324, ERR2091328, ERR2091334, ERR2091342, ERR2091342, ERR2091348, ERR2091349, ERR2091357, ERR2091358, ERR2091419, ERR2091421, DRR102690, SRR3050857, SRR3098809, SRR3987496, ERR1619552, ERR1622238, ERR1622239, ERR1622406, ERR1622406, ERR1622407, ERR1595423, ERR1543414, ERR277049, ERR1415546, ERR1163310, ERR435146, SRR2000414). All isolates were investigated for the presence of blaCMY-2 and IncK2 RNAI sequence. Positive isolates are marked by red border. All blaCMY-2 carrying isolates exhibited a p486–16-like IncK2 plasmid sequence. The allele distance between two blaCMY-2 -carrying isolates is shown. (PDF 580 kb) [file 12864_2018_4976_MOESM2_ESM.pdf]

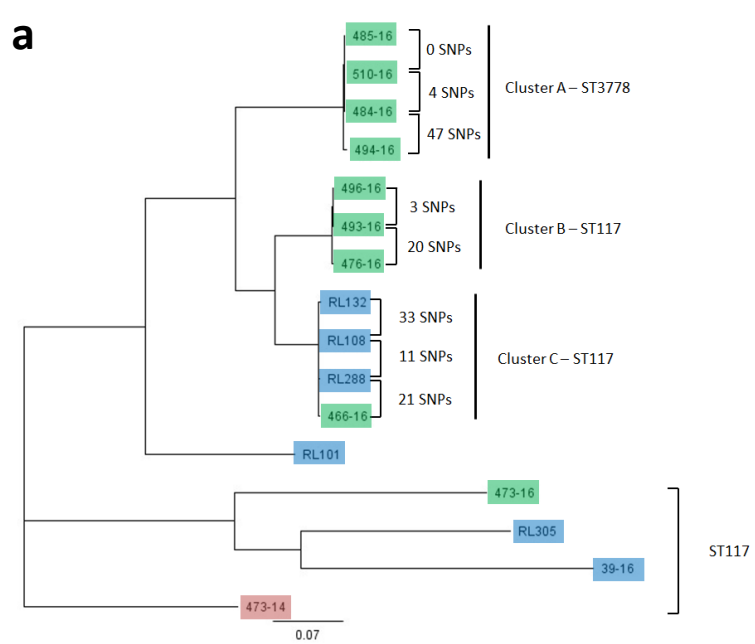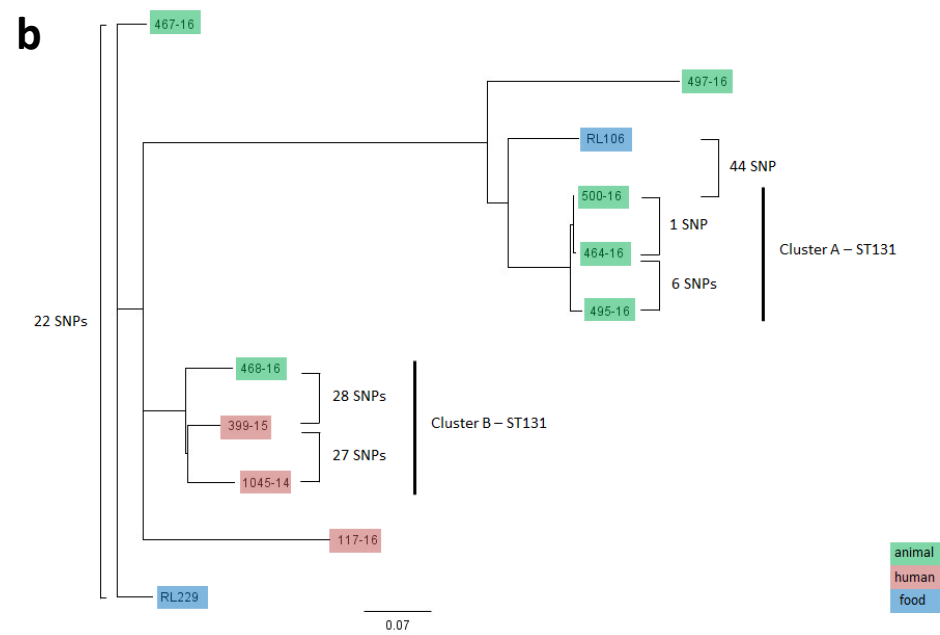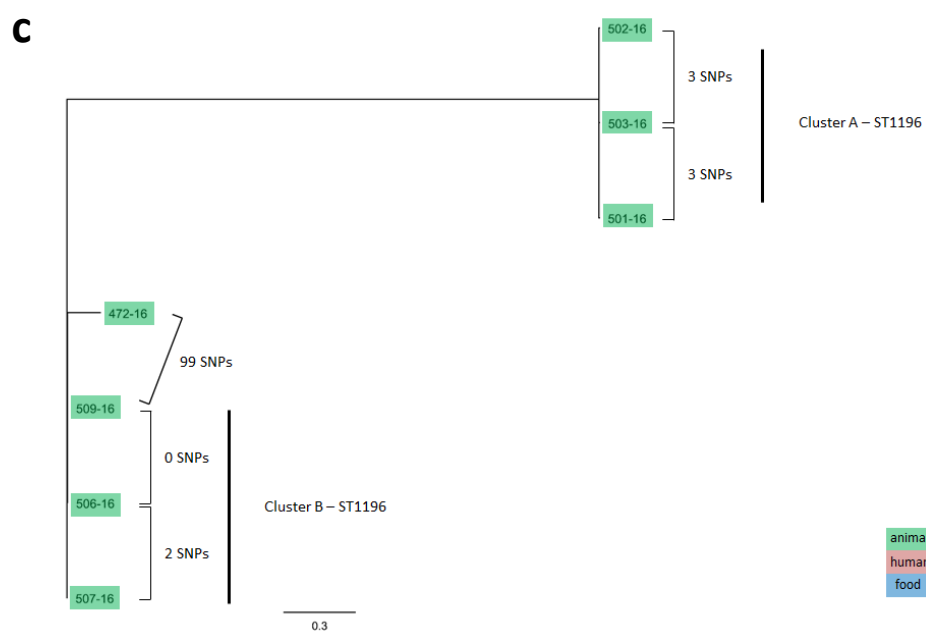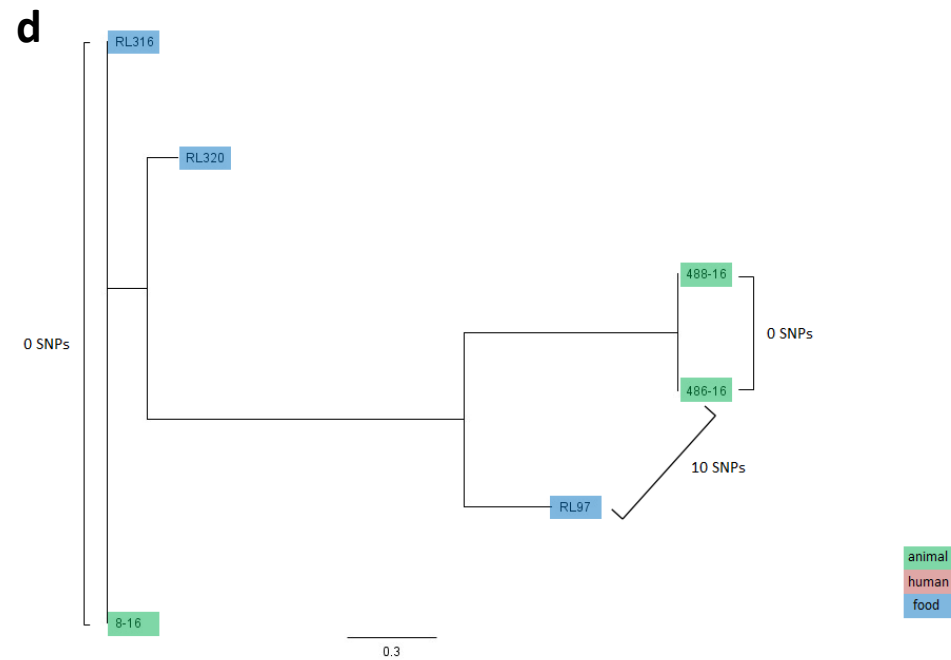

Supplement: Supplementary file 3 — Figure S2. SNP-based maximum-likelihood-trees of isolates of selected sequence types. a: Maximum-likelihood-tree of ST117 and ST3778 isolates. SNPfilter (d = 0, reference: NZ_CP019903.1 E. coli strain MDR_56) based tree, calculated with RAxML, GTR Gamma and rapid hill-climbing and 100 starting trees. b: Maximum-likelihood-tree of ST131 O25b:H4 fimH22 isolates. SNPfilter (d = 0, reference: NZ_CP019008.1 E. coli strain Ecol_AZ159) based tree, calculated with RAxML, GTR Gamma and rapid hill-climbing and 100 starting trees. c: Maximum-likelihood-tree of ST1196 isolates. SNPfilter (d = 0, reference: NC_020518.1 E. coli str. K-12 substr. MDS42) based tree, calculated with RAxML, GTR Gamma and rapid hill-climbing and 100 starting trees. d: Maximum-likelihood-tree of ST429 isolates. SNPfilter (d = 0, reference: NC_013654.1 E. coli strain SE15) based tree, calculated with RAxML, GTR Gamma and rapid hill-climbing and 100 starting trees. (PDF 174 kb) [file 12864_2018_4976_MOESM3_ESM.pdf]

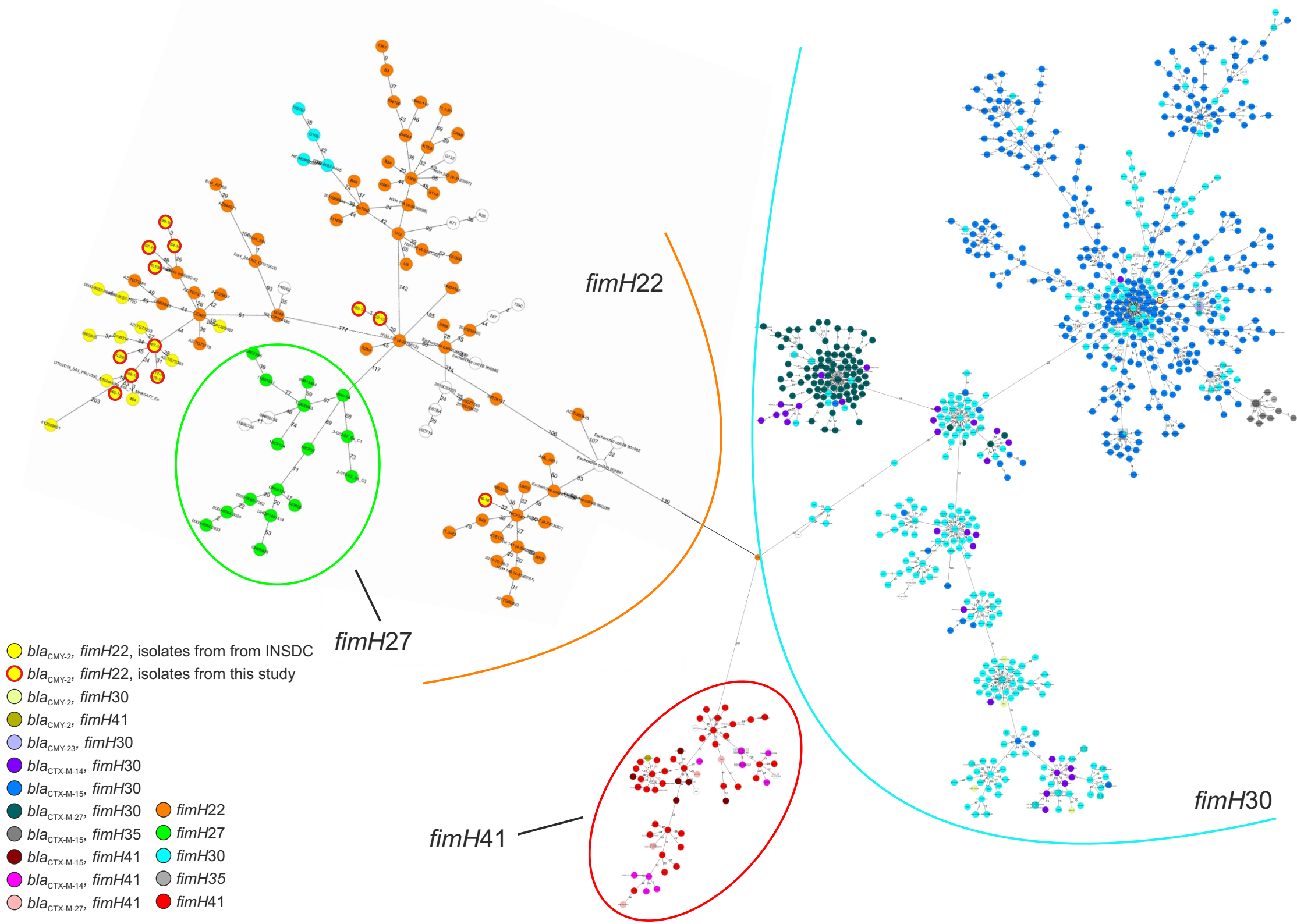

Supplement: Supplementary file 4 — Figure S3. Minimum spanning tree of E. coli ST131 isolates from this study and INSDC and Enterobase based on an ad-hoc cgMLST including 2547 alleles. All fimH alleles and blaCMY and blaCTX-M genes are color-coded. Isolates from this study are marked with a red ring. (PDF 971 kb) [file 12864_2018_4976_MOESM4_ESM.pdf]

# Inck2 plasmid variants

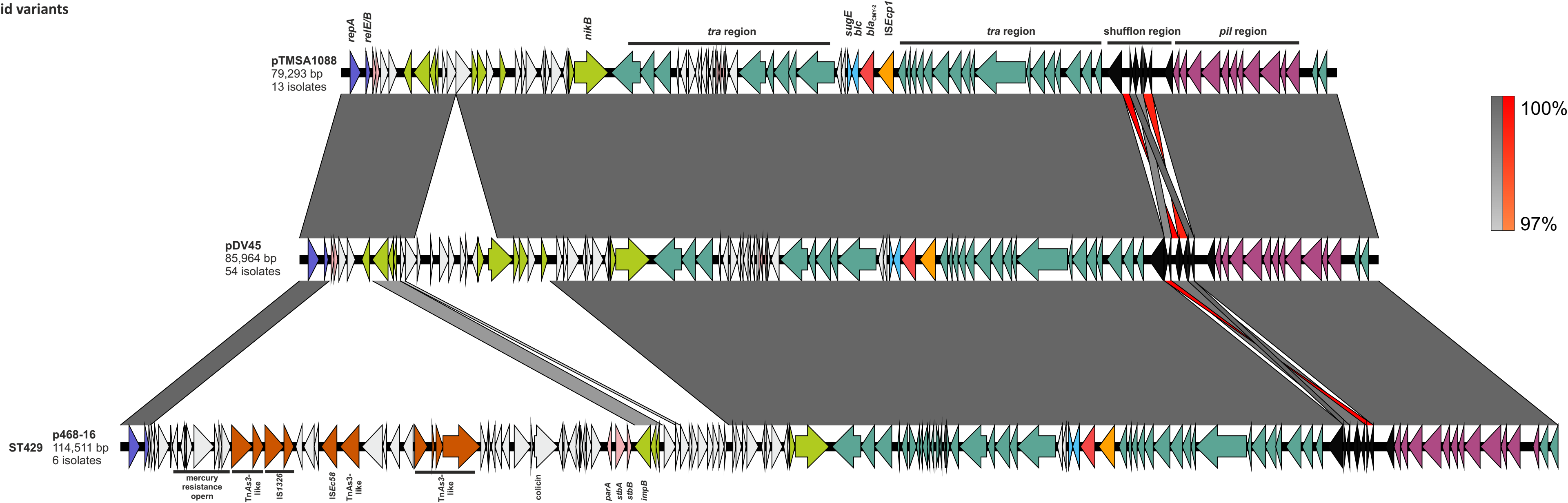

Supplement: Supplementary file 5 — Figure S4. Comparison of plasmid sequence p486–16 with other IncK2 plasmids created with EasyFig v.2.2.2 (http://mjsull.github.io/Easyfig/). Used reference plasmid sequence were pTMSA1088 (Genbank: KR905386.1), pDV45 (KR905384.1). (PDF 38 kb) [file 12864_2018_4976_MOESM5_ESM.pdf]
